# The PRotective Effect of Maternal Immunisation on preTerm birth: characterising the Underlying mechanisms and Role in newborn immune function: the PREMITUR study protocol

**DOI:** 10.3389/fimmu.2023.1212320

**Published:** 2023-12-20

**Authors:** Michelle L. Giles, Stephen Cole, Jessica O’Bryan, Sushena Krishnaswamy, Rym Ben-Othman, Nelly Amenyogbe, Mary-Ann Davey, Tobias Kollmann

**Affiliations:** ^1^ Department of Obstetrics and Gynaecology, Monash University, Melbourne, VIC, Australia; ^2^ Department of Infectious Diseases, University of Melbourne, Melbourne, VIC, Australia; ^3^ Department of Obstetric Medicine and Maternal Fetal Medicine, Royal Women’s Hospital, Melbourne, VIC, Australia; ^4^ Department of Obstetrics and Gynaecology, Epworth Healthcare, Melbourne, VIC, Australia; ^5^ Department of Infectious Diseases, Monash Health, Melbourne, VIC, Australia; ^6^ Department of Paediatrics, Telethon Kids, Perth, WA, Australia

**Keywords:** pregnancy, vaccination, preterm birth, influenza, pertussis

## Abstract

Maternal immunisation, a low cost and high efficacy intervention is recommended for its pathogen specific protection. Evidence suggests that maternal immunisation has another significant impact: reduction of preterm birth (PTB), the single greatest cause of childhood morbidity and mortality globally. Our overarching question is: how does maternal immunisation modify the immune system in pregnant women and/or their newborn to reduce adverse pregnancy outcomes and enhance the newborn infant’s capacity to protect itself from infectious diseases during early childhood? To answer this question we are conducting a multi-site, prospective observational cohort study collecting maternal and infant biological samples at defined time points during pregnancy and post-partum from nulliparous women. We aim to enrol 400 women and determine the immune trajectory in pregnancy and the impact of maternal immunisation (including influenza, pertussis and/or COVID-19 vaccines) on this trajectory. The results are expected to identify areas that can be targeted for future intervention studies.

## Introduction

Globally, preterm birth (PTB) remains one of the most significant causes of death and morbidity in childhood (1, 2). PTB is the single most important cause of death in children under five years of age, with one million children dying each year due to PTB-related complications (1). It is also an important cause of disability and major contributor to health care expenditure (3). Most PTB and stillbirths occur in low-income settings, with over 75% of cases occurring in Asia and sub-Saharan Africa, and morbidity and mortality rates of premature infants in these settings remain disproportionately high (1). Despite efforts to reduce PTB worldwide, rates of this adverse pregnancy outcome remain persistently elevated, independent of income setting (4, 5). Hence, there is an urgent need for more effective interventions to reduce PTB and its attendant neonatal and early childhood morbidity and mortality, particularly in low- and middle-income countries (LMICs).

Maternal vaccines are a well-established strategy used for prevention of pathogen-specific disease in mothers and infants through transplacental transfer of maternal antibodies. Routine tetanus toxoid-containing vaccination was the first routine immunisation program implemented for pregnant women (6). Beyond pathogen-specific benefits, some studies have also identified pathogen-agnostic or non-specific effects (NSE) of maternal vaccination protecting against several adverse birth outcomes, including PTB and stillbirth (7–11). Our group have previously published a systematic review of the safety of influenza vaccination in pregnancy (12). An unexpected finding of our review of the literature was an association between maternal immunisation and lower rates of PTB (12). However, this finding has not been consistent across other published studies. Nonetheless, we were intrigued by the possibility of immunisation being associated with reduced PTB and thus proceeded to explore this association in a large dataset of 270,851 women who gave birth in the Australian state of Victoria between Jan 2015 and Dec 2018 (11). We found that women receiving influenza vaccine and/or pertussis containing vaccine during pregnancy had a significantly lower risk of PTB than unvaccinated women (11). Further, the reduction in PTB we observed in association with maternal vaccination remained significant and consistent across four different influenza seasons (2015-2018) and across different times of the year (during and outside of influenza seasons) (11). The benefits therefore were unlikely related to preventing maternal infection or actual exposure to influenza virus. We have further confirmed this association when accounting for immortal time bias (13).

The term pathogen-agnostic or NSE of vaccination aims to capture protection against outcomes unrelated to prevention of the intended vaccine preventable disease (14). We will use the term NSE for the remainder of this article. These could theoretically be associated with any vaccine. A systematic review in 2016 sponsored by the World Health Organization concluded that the Bacille Calmette-Guerin (BCG) vaccine and measles vaccine were associated with mortality benefits greater than expected based on pathogen-specific effects alone (15). For example, although *Mycobacterium tuberculosis* is the intended target pathogen of BCG vaccines, BCG vaccination of newborns reduces risk for death from a wide range of infections unrelated to tuberculosis, including neonatal sepsis (16, 17). Mechanisms that have been postulated include changes in innate immunity, including a rapid increase in neutrophils (18).

Although the causes of PTB are not entirely understood, there are several risk factors and mechanisms that are known to be implicated in the process (19). Some studies suggest that in the case of preterm labour, multiple factors contribute to dysregulated inflammatory processes that result in early initiation of labour (19, 20). This trajectory, down an unfavourable path, may be underpinned by activity of the immune system- the so called “immune trajectory” in pregnancy. One hypothesis for the NSE of maternal immunisation on birth outcomes is that maternal vaccines may modulate the maternal and fetal immune trajectory such that these heightened inflammatory processes that contribute to PTB are reduced. Similar to the benefit of BCG on newborn host defence, maternal vaccination could for example activate innate immunity providing broad protection against pathogens, or enhance immune regulatory pathways, which together could reduce potentially harmful inflammatory responses during pregnancy (21, 22).

NSE of vaccination are increasingly being recognised for their health benefits in postnatal life. Yet, this has never been explored in prenatal life, i.e. during pregnancy. Our and others’ data indicate that maternal immunisation may increase immune resilience leading to longer gestation, and in turn, a healthy start to life. For the purpose of this study, we define “immune resilience” as the capacity to preserve and/or rapidly restore immune function that resist adverse outcomes and control other causes of inflammatory stress. Based on the above observations, and the clear need to understand mechanisms for other vaccines and interventions to tackle the substantial burden of adverse pregnancy outcomes, we designed the PREMITUR study.

## Aim and hypothesis

The PREMITUR study hypothesises that maternal immunisation alters the immune trajectory in pregnant women in a way that leads to a reduction in adverse pregnancy outcomes. The specific aims of our study are:

Aim 1. To determine the immune trajectory of pregnancy that leads to term versus PTB.
Aim 2. To define the impact of maternal immunisation on the immune trajectory of pregnancy.
Aim 3. To delineate the impact of maternal immunisation on the immune trajectory of the infant.

## Rationale

Pregnancy induces rapid changes in systemic maternal immunity. In healthy term pregnancy these changes follow a healthy developmental trajectory (23–25), while the final common pathway leading to adverse pregnancy outcome appears to involve an altered immune trajectory (26). However, the immune trajectory of pregnancy in relation to adverse pregnancy outcomes has not been comprehensively captured via the powerful integration across the range of biological domains that we will conduct here (RNA, protein, metabolite, function – ‘multi-omics’). To our knowledge, there is only one recent publication that applied a similar integrated, multi-omic approach to PTB (27). While this published report clearly confirms feasibility and advantages of our systems biology approach, the previous study only assessed a single time point in a cross-sectional design, i.e. they did not attempt to determine the trajectory (27). And importantly, this published report did not take the impact of maternal immunisation into account. We have recently shown that integrating across the multi-omic space and conducting these assessments longitudinally over time in the same subject allows delineation of developmental trajectories that provide critical insight into complex biological processes (18, 28). Importantly, this integration across biology (multi-omics) and over time in the same subject allows for sound and rigorous statistical interrogation of group sample sizes with n ≤20 (18, 25).

Our group has also recently shown that one’s “immune baseline” can determine the outcomes of vaccination or infection (29, 30), and that for the immune trajectory of the newborn this ‘baseline’ is influenced by maternal immunity (31, 32). We have also shown that the ‘baseline’ in early life is not a stable steady state (as it is in adults), but a dynamic yet highly predictable (and predictive) trajectory (28). This suggests that impacting the mother’s immune trajectory in pregnancy through maternal immunisation may impact the immune trajectory of the infant. The concept of maternal immunity impacting immune ontogeny of her infant is already widely accepted for maternal antibodies influencing the infant’s antibody response to vaccination (33). But there is growing evidence that this mother-infant interaction goes far beyond adaptive immunity: i) maternal quadrivalent influenza vaccine (QIV) reduces acute airway infections in the infant beyond influenza (34) which may relate to vaccine-induced cytokine responses in the nasal mucosa of her infant (35); ii) maternal diphtheria, tetanus and acellular pertussis (dTpa) immunisation during pregnancy reduces the risk for acute airway infections beyond Bordetella pertussis in infants, which may relate to vaccine-induced cytokine transfer across the placenta (36). The theoretical underpinnings of our rationale for Aim 3 are thus supported by existing evidence. What is lacking is evaluation of the maternal-infant immune dyad in the context of maternal immunisation using the unbiased, systematic approach of systems biology proposed here. Our findings promise not only to unveil the mechanisms related to maternal immunisation but to identify pathways to target to improve the developmental trajectory and well-being of all children in general.

## Methods and analysis

### Study design

This is a prospective, observational cohort study.

### Study sites and sample size

There are four maternity services participating in this project- two private and two public. Together these services have over 25,000 births per year, of which half are primigravid women.

### Private health service partner involvement

In this project we partner with both public and private providers of antenatal care. In Australia almost one quarter of births occur in the private sector although this group is rarely included in research. It is essential to consider the private sector to maximise the opportunities for translation of key findings and to ensure broad representation of women so findings can be generalizable.

### Consumer and community involvement

Miracle Babies is Australia’s leading organisation supporting premature and sick newborns, their families and the hospitals that care for them. They are partnering on this project and will be providing input end-to-end for this study. This includes providing consumer representation on the project steering committee, design and review of the study protocol, review of the conduct and progress of the study, interpretation of the data and most importantly appropriate and sensitive dissemination of the findings.

### Inclusion criteria

A pregnant woman will be eligible for inclusion in the PREMITUR study if the following criteria are met

- Nulliparous
- Aged 18 to ≤45 years
- Gestation between 9 and 22 weeks at time of recruitment
- Willing and able to comply with all study requirements including timing and/or nature of required assessments and follow up of mother and baby to six weeks post-partum

### Exclusion criteria

A pregnant woman will be ineligible for inclusion in the PREMITUR if the following criteria apply:

- Multiparous
- Multiple pregnancy
- Risk factors for PTB e.g. previous cervical procedure such as cone biopsy
- Unable to provide consent
- Concomitant immunosuppressive medication likely to continue beyond first trimester
- Any other significant acute or chronic medical condition that in the opinion of the investigator may interfere with the interpretation of study results or place the participant at increased risk if they participate in the study.
- Unwilling and unable to comply with all study requirements including timing and/or nature of required assessments and follow up of mother and baby to six weeks post-partum (e.g. planning to relocate during pregnancy)

### Recruitment and procedures

We aim to recruit 400 healthy nulliparous women over three years. We are restricting our recruitment to primigravid women.

**Figure 1:**
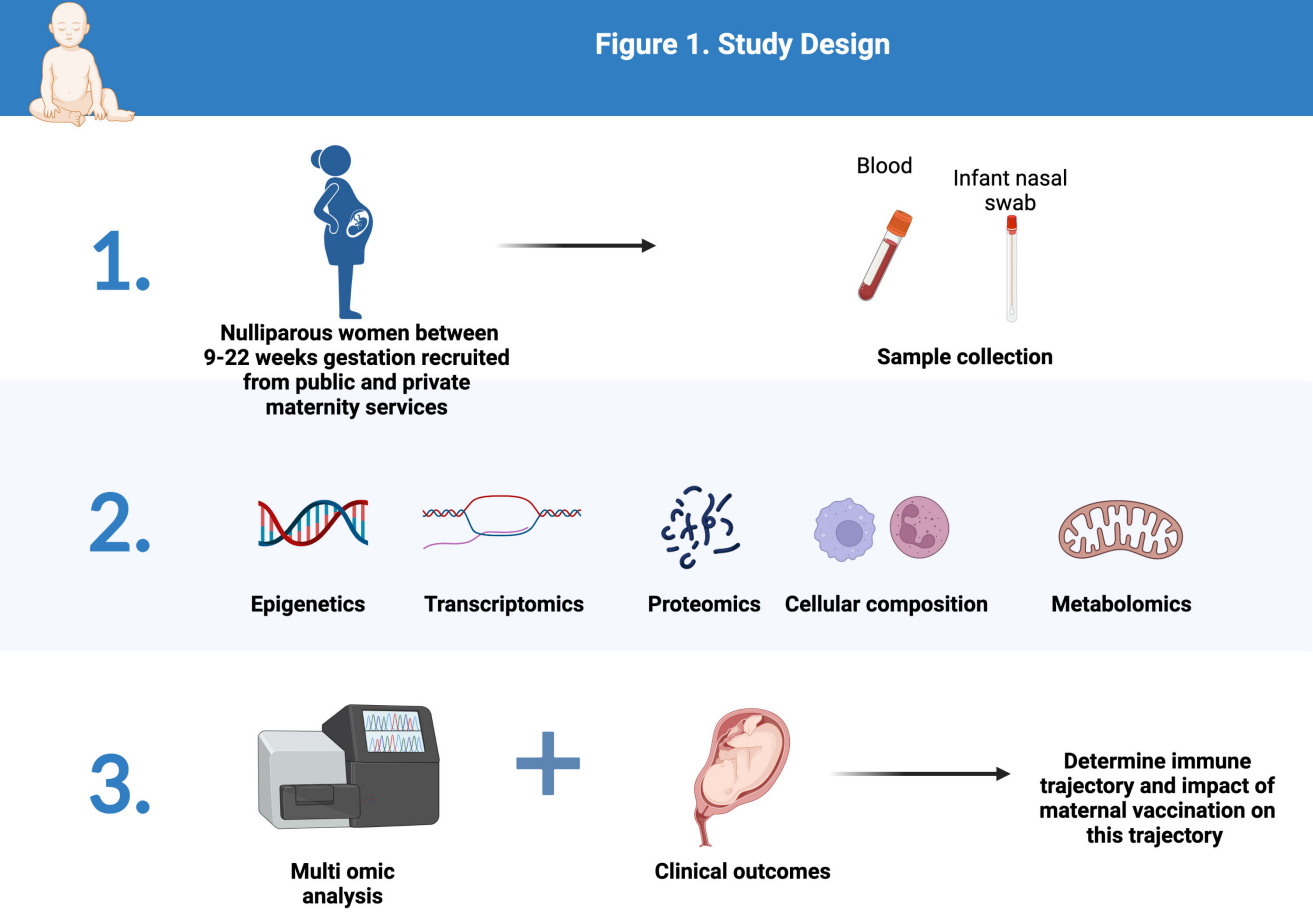
Study Design (Created with BioRender.com).

Potential participants will be identified by one of two mechanisms. In the public system women will be identified from clinic lists as eligible by a research midwife who will alert the treating clinician. If deemed appropriate the treating clinician will introduce the study to the participant and seek their interest. If they are interested to receive further information then their details will be shared with the research midwife who will contact them, provide further information, answer questions and provide the participant information and consent form (PICF). Signed consent can be obtained either electronically or in paper form with a wet ink signature. A similar process is followed for women receiving private maternity care, although in this case identification of eligible women is via the private obstetrician (**Figure 1**).

**Figure 2:**
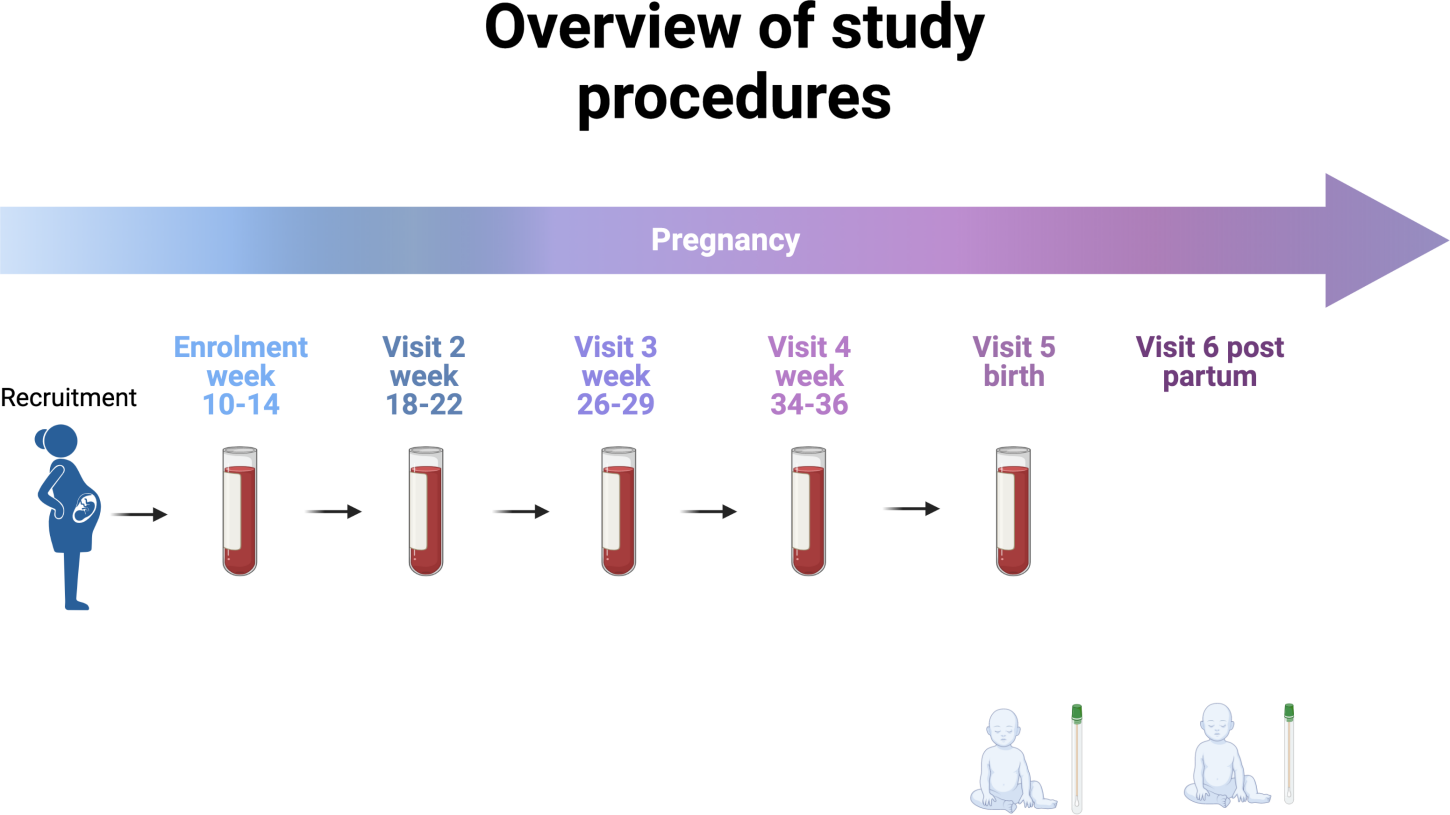
Overview of study procedures (Created with BioRender.com).

Clinical data will be collected at baseline including established risk factors for PTB as well as past vaccination history. Pregnancy outcomes will be collected at birth. Venous blood (5mL) will be collected at each timepoint: 10-14 weeks, 20-22 weeks, 26-28 weeks, 34-36 weeks, and at birth (**Figure 2**).

We have chosen these time points to afford serial profiling while minimising burden and coinciding with routine pregnancy care appointments.

### Sample collection, processing, storage and data generation

Specifically, blood will be collected for transcriptomic (RNASeq) as well as epigenetic (methylation) analysis and plasma harvested for proteomics (via Somalogic Inc.) and metabolomics (via Metabolon Inc.). Cell composition of this blood sample will also be assessed by flow cytometric analysis capturing most innate and adaptive immune subsets, from neutrophils, dendritic cells (DCs) and their subsets, to NK cells, monocytes, as well as most T cell subsets and B cell subsets. The Paxgene tube will be used for transcriptomics. As little as 55 microlitres of blood is required for proteomics and metabolomics, so 5mL per timepoint is sufficient for the proposed testing. Given the longitudinal nature of our study set up, samples will be batch randomized to reduce artifact, as well as corrected using ComBat batch to normalise across multiple analytical runs. Following distributional normalisation, we will apply the full power of longitudinal samples from the same participants, i.e., features will be normalised as change over time within each woman, using the WithinVariation function implemented in the R *mixOmics* package. With this, absolute values will be represented as relative change over time - i.e. biological trajectories. We will analyse multiomic signatures across women to define the immune trajectory of pregnancy. We will then contrast omic signatures across clinical groups (healthy term vs. PTB).

Assessment of the newborn infants’ response to maternal immunisation will focus on the nasal mucosa of the infants born to mothers enrolled in our study. Samples will be obtained on day of life 1 and at 6 weeks of age via a standardised, non-invasive and painless sampling procedure (Nasosorption), well-tolerated by newborn infants (and their parents) (37). These time points were chosen to coincide with infant health checks to reduce additional burden to parents. The neonatal nasal lining fluid absorption devices will be used to extract total proteins contrasting the infants’ developmental trajectory as a function of maternal immunisation status. The method of isolation of total proteins from the nasal lining fluids starts by thawing the devices on ice to preserve the integrity of the cells. Then, 300 ul of elution buffer (PBS (pH7.4) containing 0.05% (v/v) Tween20) is added. The synthetic absorptive matrix (SAM) is then removed from the nasosorption device using forceps by tearing it from the handle using sterile forceps. The SAM is then placed into a spinX column using a new pair of sterile forceps. After a centrifugation of the column and SAM at speed centrifugation(16,000 g) for 30 minutes at 4C, 260 ul of equate is reconstituted into a sterile tube and total proteins are quantified using Pierce BCA protein assay kit The protein identification and quantities will be assessed using Somascan, an untargeted Proteomics platform (Somalogic) capable of detecting more than 7000 proteins in less than 200ul of eluate (200ug/ml). In the testing assays performed by the study team, enough proteins were extracted from infant nasal lining devices and only a portion was used for proteomics analysis. The rest of the samples can be stored and used for targeted protein detection panels if interesting signals are detected from the initial untargeted analysis.

Our group and others have proven that when using this integrated multi-omic approach a sample size of 20 per group will suffice to identify robust (80% power) biological signatures related to pregnancy outcome with a two-sided significance criterion (alpha) of < 0.05. With this in mind, a sample size of 400 participants was chosen, assuming a background rate of PTB of 8%, it was estimated 32 women would delivery prematurely providing >20 per group.

#### Analysis plan

##### Aim 1

We will compare the immune trajectory of all women with a preterm birth (estimated to be 8% of our total sample size) with a matched control group of women who deliver at term. We will not be performing multiomics on all participants recruited. To assess the immune trajectory in relation to pregnancy outcome longitudinally over time we will identify features both in the univariate and multivariate space. For univariate analysis, differentially expressed (DE) genes, proteins, or metabolites will be identified using DESeq2 v1.26. Correction for multiple comparisons will be done to control for false discoveries, filtering results based on a highly conservative p-value for significance (<0.001). Sparse Partial Least Squares - Discriminant Analysis (sPLS-DA) will be used to identify discriminatory features that co-vary with each other. Given the user-defined sparsity feature of sPLS-DA, and with the readout being classification accuracy, no p-value adjustment is necessary for this method. To assess the data in their biological context, i.e. integrated multi-omics, we will use two complementary integration strategies. *NetworkAnalyst* is based on known protein-protein interaction (PPI) networks and *DIABLO*, part of the *mixOmics* framework, a data-driven multi-omic integration approach. Given that *DIABLO* selects features that allow maximum class distinction (i.e. accurate classification of pregnancy outcome), this approach will also identify the earliest point during pregnancy that can accurately predict an adverse birth outcome. Finally, time course analyses that generate smoothing splines allow identification of groups of features that follow the same trajectory in a multivariate space. The *DynOmics* toolbox is an extension of the *mixOmics* toolbox used for multi-omic datasets built to identify these dynamic longitudinal changes in biological systems, while integrating multi-omic data.

##### Aim 2

We will compare the immune trajectory of 20 unvaccinated women with 20 women who received either one of the two vaccines recommended in pregnancy, and 20 vaccinated with both vaccines. Recruitment started prior to the availability of COVID-19 vaccines but continued during the rollout of COVID-19 vaccines. Data on any vaccination during pregnancy is captured and will be taken into account in the analysis. Based on our previous work, we expect ~10 women in our cohort to choose to not be vaccinated at all, and approximately 50 women to be vaccinated with either influenza or pertussis alone. If we are unable to recruit 10 controls who have received no vaccination we intend to use women who are vaccinated late in pregnancy as a control over the time periods they remain “unvaccinated”. We will employ the same blood processing and data analysis pipeline summarised for Aim 1.

##### Aim 3

Samples will be obtained on day of life 1 and 6 weeks of life (day of recommended first follow up) via our highly standardised, non-invasive thus non-painful sampling procedure (Nasosorption), well-tolerated by newborn infants (and their parents) as described. We will process and analyse these 120 samples as described above contrasting the infants’ immune trajectory as a function of maternal immunisation status.

## Data capture, quality control and management

### Clinical data

Clinical Data will be collected across all study sites using REDCap, a secure electronic platform with built-in quality checks and access to the platform is limited to the study teams with clinical good practice up-to-date certificates, and unique credentials are required to log in.

To further ensure the quality of our data, we will be implementing quality assurance (QA) processes. These processes will be performed by the study team, and any discrepancies or issues that arise will be raised and resolved before sharing the data across the project analysis teams. Once the study is complete, the database will be locked, and data downloads will be restricted to read-only access.

### Laboratory data

In addition to clinical data, our study will also be collecting laboratory data from all study sites. MAESTRO (Multiomic Analytics, Experiments, and Sample Tracking Orchestrator) via RAN BioLinks, Ltd., a sample tracking platform will be used and access granted using specific credentials for each study team. MAESTRO is a modular platform that allows us to track and manage operational data in the laboratory, sample inventory, storage, and processing sites information as well as information about study samples, such as the collector, time, barcodes, volume, and any deviation from the protocol. It also enables easier integration with de-identified clinical data from REDCap in the future.

In addition to sample tracking, MAESTRO also includes a project management feature. This feature allows researchers to create centralized documentation, set goals, track milestones, manage team membership and roles attribution, and store up-to-date protocol documents. To ensure data quality, MAESTRO has a built-in a quality control layer. This layer automatically flags any inconsistencies or out-of-range data according to study-specific protocols and procedures.

### Quality control

To minimize the potential confounding effects of batch effects across different platforms, we will be aligning our sample batches for all “OMIC” platforms with the study block randomization. This approach ensures that we include study samples from the same participant across time points and representation of samples from different groups within each batch.

Each assay platform used in our study has predefined criteria for quality control of each experimental sample and assay. At a minimum, internal controls and standards will be run across all runs to ensure data quality.

### Confidentiality

Finally, to ensure confidentiality, all electronic data capture will be performed using secure, password-protected, encrypted devices. Paper records, including consent forms and medical records, will be stored in locked cabinets in secure locations with restricted access at the study sites. By taking these measures, we can ensure that our study meets the highest standards of data quality and confidentiality.

### Ethics and dissemination

This trial was reviewed and approved by the Monash Health Human Research Ethics Committee (HREC). The trial is also registered on the Australian and New Zealand Clinical Trials Registry (ANZCTR). The trial will be conducted in compliance with local and National Health and Medical Research Council (NHMRC) guidelines. The outcomes of the clinical trial will be disseminated through local, national and international meetings and in peer-reviewed medical journals. Authorship will be allocated to final publication for study investigators and those providing substantive contributions to the design, conduct, interpretation, and reporting of the clinical trial.

## Discussion

In the ten minutes it has taken to read this manuscript, almost 300 more babies will have been born prematurely around the world. This global health problem needs urgent and innovative approaches. This project will capture for the first time the biological pathways responsible for protection against adverse pregnancy outcomes and prevention of illness in infants through increased immune resilience during pregnancy. This would confirm the place of maternal immunisation as part of a “three for one” deal including pathogen-specific protection for the mother and neonate, and non-specific immunological benefits leading to protection for the fetus. Finally, our work will empower women to base their choices around vaccination during pregnancy on evidence and insight. Specifically, our project not only has the potential to unlock mechanisms that underpin the beneficial effect of maternal immunisation, but provide immediate translational avenues, as all vaccines under study are already licensed and proven to be safe in pregnancy.

## Author contributions

MG and TK conceived the concept for this study. MG, SC, JO, SK have contributed to study design and recruitment. TK, M-AD, RB-O and NA have contributed to methodology and planned analysis. All authors have contributed and reviewed the protocol and this manuscript. All authors contributed to the article and approved the submitted version.
